# Comprehensive Model for Mental health Access and service use (CoMMA): A process model for technology-enhanced mental healthcare

**DOI:** 10.1016/j.invent.2026.100927

**Published:** 2026-02-27

**Authors:** N.A.J. De Witte, P. Best, J. Torous, M. Mulvenna, E. Van Assche, K. Mathiasen, A.M. Kleiboer, P. Carlbring, T. Van Daele

**Affiliations:** a Psychology & Technology, Centre of Expertise Care and Well-being, Thomas More University of Applied Sciences, Antwerp, Belgium; b TIME Centre for Technological Innovation, Mental Health and Education, Queen's University, Belfast, United Kingdom; c Division of Digital Psychiatry, Beth Israel Deaconess Medical Center, 330 Brookline Ave, Boston, MA, 02215, United States; d School of Computing, Ulster University, Belfast, United Kingdom; e Dept. of Psychology and Behavioural Sciences, Aarhus University, Denmark; f Dept. of Clinical Research, University of Southern Denmark, Denmark; g Department of Clinical, Neuro, and Developmental Psychology, Amsterdam Public Health Research Institute, Vrije Universiteit Amsterdam, Amsterdam, the Netherlands; h Department of Psychology, Stockholm University, Sweden; i School of Psychology, Korea University, Seoul, South Korea

**Keywords:** Mental health, Digital mental health, Treatment, Blended care, Self-help, Healthcare systems

## Abstract

Over recent decades, mental healthcare reforms have been proposed to facilitate deinstitutionalization, integration into primary care, task-sharing to non-specialist providers and, more recently, digital interventions. All are aimed at improving the accessibility, acceptability and effectiveness of care. However, many healthcare systems still suffer from complexity, rigidity and inequity in access. New and more integrated models of service delivery are needed to fully harness the potential of evidence-based approaches in mental healthcare, including digital or community-based interventions. The current contribution provides an overview of recent developments - in the organization of care, allocation of healthcare services, and the digital transformation of care - and presents the Comprehensive Model for Mental health Access and service use (CoMMA). The process model includes both informal support (e.g., self-help and community care) as well as formal services (e.g., diagnostics and interventions delivered by healthcare professionals). In line with the increasing digital transformation of care, CoMMA also addressed how technology can play a role in the different model components. The purpose of the model is to provide guidance to healthcare systems, professionals and trainees in shaping the provision of evidence-based psychological services and implementing interventions. It hereby aims to complement ongoing societal, regulatory, and economic changes in the healthcare field by providing a conceptual and substantive narrative. The model shows how mental health services can be organized based on current scientific frameworks, policy perspectives, and clinical practice.

## Highlights

• The Comprehensive Model for Mental health Access and service use is presented.
• CoMMA aims to support professionals in shaping mental health services.
• Digital transformation including care reorganization can improve mental healthcare.
• Systems struggle with care provision and new service delivery models are needed.

## 1 Introduction

While over one billion individuals are affected by mental ill health worldwide, mental healthcare systems struggle to provide timely and appropriate access to support and care (World Health Organization, 2025). For example, treatment coverage for mental health service use for major depressive disorder ranges from an average of around 30% in high-income locations to less than 10% in low- and lower middle-income countries (Moitra et al., 2022). While medication may be the most accessible and common intervention for mental health problems, social and (digital) psychological interventions are also effective for prevention and care (Hedman-Lagerlöf et al., 2023) and the field has been observing a shift from the medical model of mental illness toward more individualized psychological and social models (Patel et al., 2023). Over recent decades, mental health systems have been restructured through deinstitutionalization, integration into primary care, task-sharing to non-specialist providers and, more recently, digital interventions, all aimed at improving the accessibility, acceptability and effectiveness of care (WHO, 2025; Patel et al., 2023). While mental healthcare is currently facing major challenges, several opportunities also arise to critically analyze and reorganize mental health services. The Comprehensive Model for Mental health Access and service use (CoMMA) aims to provide a substantive process model for healthcare delivery by building on developments along three dimensions: the organization of care, allocation of healthcare services, and the digital transformation of care. We will summarize the relevant developments in these three dimensions since they have laid the foundation for the CoMMA model, which is described in the next section.

### 1.1 The organization of care

The narrative for the treatment of mental illness has for a long time centered around institutional care, psychotherapy, and pharmaceutical treatment. However, during the last decades, there has been a shift from traditional siloed care to blending mental health within general medical care at the system level. This has been termed **Integrated (Mental Health) care** (with a strong emphasis on integrated primary care). Two important frameworks capitalizing on integrated care are the Collaborative Care Model and the Model for Optimal Mental Health Care from the World Health organization. The **Collaborative Care Model (CCM)** refers to an approach where a multidisciplinary team provides mental health services in primary care. It adopts patient-centered team-based care, which refers to a collaboration between patient and professionals – including primary care providers, care managers, and mental health clinicians (Lim et al., 2024). Other important components of collaborative care are (1) the provision of evidence-based care, (2) measurement-based treatment such as using registries to ensure patient improvement (Hu et al., 2020), and (3) population health management to support equity (Jackson-triche et al., 2020).

The World Health Organization's (WHO, 2009) **Model for Optimal Mental Health Care** advocates for integrated primary care and emphasizes the importance of both formal and informal community-based mental health services. The service organization pyramid for optimal mental health care consists of five layers with a decreasing frequency of needs and increasing costs. Self-care is both the base of the service organization pyramid and an additional dimension that coincides with other mental health services. In 2022, the WHO even released a new guideline specifically for self-care interventions for health and wellbeing (WHO, 2022). The second tier consists of informal community care, which refers to supportive, counseling or self-help services and individuals in the community, such as teachers and family associations, but in certain contexts also traditional healers or NGOs. The following three tiers are components of formal healthcare. The third tier of primary care mental health services includes professionals providing assessment, mental health promotion, basic psychosocial services, and referral. The fourth tier consists of psychiatric services in general hospitals and community mental health services. The top tier refers to long-stay facilities and specialist psychiatric services.

Non-specialist practitioners and community members can deliver effective community-based psychosocial interventions in a collaborative care delivery model (Patel et al., 2023). Previous research has also underscored the importance of informal help and good support networks, e.g., for young people (Zhao and Hu, 2022; Ishikawa et al., 2023). In this respect, it is important to acknowledge that each individual is embedded in a context which can promote or threaten their mental health. The WHO's framework for the **social determinants of health** aims to provide insight into how the structure of societies affect population health (Solar and Irwin, 2010). It indicates that context-specific interventions and policies can target the “micro” level of individual interactions (e.g., households or workplaces) but also the “*meso*” level of community conditions or the “macro” level of universal public policies and the global environment. This is in line with other theories identifying multiple concentric physical and virtual systems around individuals, which influence health and wellbeing (e.g., Navarro and Tudge, 2022).

While community care and self-help have been identified as effective approaches and valid ways to reduce pressure on formal healthcare, both approaches are struggling with large-scale implementation as evidenced by the underfunding of community-based mental health approaches (Patel et al., 2023; WHO, 2025) and substantial variability in uptake of self-help (Baumel et al., 2019; Lipschitz et al., 2022).

### 1.2 Allocation of healthcare services

Individuals can gain access to mental health services in different ways. The **Model of Help-seeking** by Rickwood and colleagues views the process of seeking support as a multi-stage non-linear process which starts with an individual experiencing certain symptoms and becoming aware of a need for help (Rickwood, 2022; Ishikawa et al., 2023). As a next step, intentions are being shaped as individuals identify help sources and reflect on their willingness or readiness to engage with them. Help sources can be formal (e.g. professionals), informal (e.g., friends), or self-help (e.g., online information seeking). Finally, we can observe actual help-seeking behavior to address the problem, where the individual approaches a help source and engages. However, help-seeking activities might seize in earlier steps and therefore result in not accessing any help sources. While access to mental healthcare can still require a referral from a general practitioner, systems increasingly also include the possibility of self-referral (Gunn and Flehr, 2023). Self-referral has been linked to better treatment effects of digital mental health interventions, possibly due to higher motivation and experienced autonomy (Bjarke et al., 2025).

Within mental healthcare, two approaches can be used to sequence intensity of care. The **Stepped Care Model** (SCM) of healthcare delivery focuses on providing individuals with interventions which are expected to provide significant health gain but are also the least intensive or restrictive option (Mareya et al., 2024). When patient needs evolve or current interventions do not lead to adequate outcomes, patients can ‘step up’ to a different intervention option (e.g., upwards in the WHO model for optimal care; WHO, 2009). The SCM model was designed to balance effectiveness with accessibility and resource allocation, and can capitalize on the increase of evidence-based digital low-intensity interventions (e.g., self-help) to foster therapeutic outcomes (Mareya et al., 2024). A practice example of stepped care is the ‘Improving Access to Psychological Therapies’ (IAPT) program in the UK (now called NHS Talking Therapies, for anxiety and depression), which set out in 2010 to deliver psychological care in primary care settings using stepped care. While the program, including a self-referral approach, significantly increased access to psychological services and showed reliable improvement rates in those who engaged, it did not seem prepared for the complexity of mental health complaints in the general population and drop-out was substantial (Martin et al., 2022). There is also a lack of evidence showing that this approach reduced the need for more intensive secondary care (Martin et al., 2022). Implementation of the SCM also faces substantial challenges related to patient preferences and engagement, professional collaboration, and integration in the broader mental health system (Mareya et al., 2024). If patients repeatedly fail to meet recovery criteria in the initial, less intensive steps of an SCM before progressing to higher-intensity care, there is an inherent risk of patient demoralization, low motivation, and potentially increased dropout at subsequent treatment steps (Nordgreen et al., 2016). However, there is evidence from studies of the service in England and its variant in Norway, of economic benefits in employment and earnings for those availing of the service (Muñoz-Navarro et al., 2024; Rzepnicka et al., 2025; Smith et al., 2025).

An alternative approach is **Matched Care** where an individual would be matched to the treatment option which is most suitable for them, instead of the least intensive option in the SCM. Pre-treatment patients' characteristics can be assessed using clinician-administered decision tools to identify specialized care needs and subsequently used to identify the most beneficial level of care (Van Krugten et al., 2020). Many real-world ‘stepped care’ services do actually operate in a hybrid fashion, combining sequential stepping with **stratified elements**, whereby more complex or severe cases are triaged directly to high-intensity (‘higher step’) interventions rather than being required to start in low-intensity care (Berger et al., 2021; Delgadillo et al., 2022). This approach can draw on the benefits of both stepped and matched care.

### 1.3 The digital transformation of care

Digital technologies are gradually finding their way into the psychological and social care systems where they are being implemented to improve access to care and provide a variety of supporting services, from assessment to skills training or full online treatment (Bond et al., 2023; De Witte et al., 2021). Recently, research is proposing technology-enhanced Matched Care approaches, mostly using biomarkers to provide insight into evidence-based treatments (mostly medication in this case) to which an individual is likely to respond (Arns et al., 2023). In line with this, however, **Precision Medicine for Mental Health** builds on clinical and biological heterogeneity in ill mental health to develop and validate clinical prediction models for stratification of treatment and prevention (Koch et al., 2024). Due to interactions between genomic and environmental factors and in line with the biopsychosocial model (Bolton, 2023), Koch et al. (2024) advocate for using varying types of genotypic, clinical and lifestyle data together with big data analytics to identify health risks, risk factors for treatment response, adverse effects, and comorbidities. Increasingly, large language models are being developed to analyze these data streams, ranging from genetics to consumer wearables (Erturk et al., 2025; Xu et al., 2025).

Such an approach can adopt Digital Phenotyping**,** referring to continuous real-time data collection with smart devices, sensors and mobile apps to derive behavior as well as physiological and psychological states (Zhang et al., 2025). Clinical decision support analytics from big data could contribute to personalized treatment plans and a shift from reactive to proactive preventive care (Burns et al., 2024). Recently, AI-based patient **flow management** tools have also been presented to support healthcare organization, including patient volume forecasting and resource planning and management (Canadian Agency for Drugs and Technologies in Health, 2024). AI-enabled referral tools such as limbic.ai are already being used to match services to individuals hereby increasing access, especially for minority groups (Habicht et al., 2024; Rollwage et al., 2024). However, at present the focus of presicion medicine is still mostly on pharmacological treatment, and major challenges relating to privacy, ethics, AI guardrails, and availability of datasets should be addressed.

Apart from care matching, care delivery can also benefit from digital opportunities. The model of **Hybrid Care** combines synchronous face-to-face or telehealth contact and asynchronous digital interventions (Chen et al., 2024). This concept has also been termed **blended care** and implementations can vary greatly in terms of the content of treatment elements, clinical targets, as well as delivery processes (Chen et al., 2024; Ferrao Nunes-Zlotkowski et al., 2024). A related concept is the **Digital Clinic** model of care (Macrynikola et al., 2025). In this model, the clinician and patient have therapy sessions via telehealth, accompanied by using an app which is personalized for patients' goals (e.g., mindLAMP). A digital navigator, who is a trained coach, helps patients with onboarding and provides ongoing assistance to support engagement and to tackle technical problems. As a next step, the app is used to enable real-world skill practice and measurement-based care and data can subsequently be summarized (by the digital navigator or one day even AI (Flathers et al., 2025) in preparation for the next therapy session. Digital navigation has been put forward as a new role within (mental) healthcare, for example as part of a collaborative care team (Lim et al., 2024). A digital navigator is a member of the care team who can facilitate the integration of technology in mental healthcare by supporting digital equity, access, selection of technologies, engagement with digital interventions, and quality evaluation of digital data to improve care (Lim et al., 2024; Wisniewski et al., 2020). Digital Clinics could benefit from integration in primary care (Macrynikola et al., 2025) and can complement collaborative care models used today.

Digital interventions can be a relevant part of collaborative care since they can facilitate reach and measurement as well as contribute to efficient resource allocation. Digital transformation of services has also provided a significant boost to low-intensity and informal mental support options, for example resulting in a proliferation of research on self-help and commercial self-help applications (Fitzpatrick et al., 2024). When used in a self-guided manner, smartphone apps show some effects but engagement remains a persistent challenge (Torous et al., 2025). Mental health interventions provided by Generative AI chatbots have been proposed to boost engagement and demonstrate effectiveness, although human supervision is essential (Heinz et al., 2025). New approaches combining a digital program including AI-driven personalized content delivery with clinical support can be effective and efficient (Palmer et al., 2025).

Several governments and regulatory agencies have been leading the way to create the necessary legislative and economic preconditions for the use of digital technologies in mental healthcare. In the Nordic countries, healthcare systems have been working on embedding internet-delivered Cognitive Behavioral Therapy (iCBT) in routine care on a central level (Vernmark et al., 2024). Other examples, such as digital clinic implementations for blended care also exist (Connolly et al., 2021; Titov et al., 2018). In Germany, the Digital Care Act of 2019 recognized digital health applications as reimbursable therapeutic devices (known as the ‘Digitale Gesundheitsanwendungen’ (DiGA)), resulting in over 374,000 prescriptions, of which over 30% are related to mental health (Schmidt et al., 2024). However, DiGA implementation has faced many challenges, including delayed patient access, lack of instructions from prescribing physicians, lack of usage and efficacy tracking, and lacking sales and distribution networks for manufacturers. Schmidt et al. (2024) identified the critical importance of digital health literacy and physician's affinity toward digital technologies as well as need for greater integration in the broader health infrastructure. Advances in AI in the healthcare domain are also being monitored carefully by the US Food and Drug Administration (FDA) but they also indicate the need for a comprehensive approach spanning consumer and health care ecosystems to prevent risks and balance economic considerations with improved clinical outcomes (Warraich et al., 2025). While legislative and technical preconditions for the adoption for digital mental health can be met, digital mental health is often still considered a novelty or a specialist domain by healthcare providers and as a result rarely structurally integrated in existing care pathways and process models (Van Daele et al., 2021).

## 2 A comprehensive model for mental health access and service use (CoMMA)

**Fig. 1:**
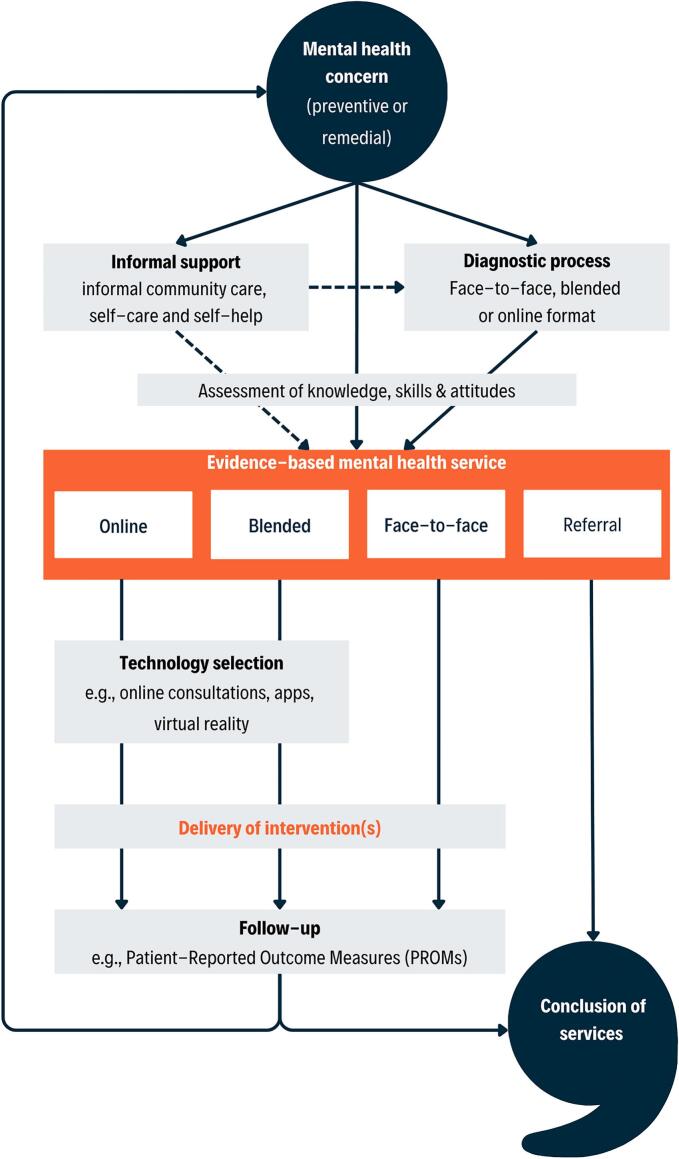
The Comprehensive Model for Mental health Access and service use (CoMMA).

Mental health systems can suffer from high complexity, rigidity and inequity, which is not in the best interest of the patient, nor the professional (Murray and Knudson, 2023). Healthcare systems would benefit from leveraging the increased diversity in access and intervention approaches. New and more integrated models of service delivery are needed to fully harness the potential of evidence-based approaches in mental healthcare, including digital or community-based interventions. We aim to describe a process model for collaborative care leveraging technology, under consideration of modern organizational pressures and treatment allocation options. The CoMMA model acknowledges current scientific evidence, policy perspectives and existing clinical practices and represents the patient journey along a digitally-enhanced mental health system (Fig. 1). In line with developments in care organization, service allocation, and digital transformation, it builds on integrated care including informal services, incorporates multiple access pathways and tailored intervention options, and integrates technology-enhanced care, respectively. It is a flexible framework which can guide (aspiring) professionals in the field, but also support thoughtful, evidence-informed implementation.

### 2.1 Mental health concern

Individuals and those in their immediate environment (e.g., GPs, relatives) may encounter mental health concerns that prompt a search for formal or informal sources of support. Such needs may fall within the domain of prevention (e.g., fostering resilience, mitigating stress) or focus on the identification and treatment of existing issues (e.g., symptoms of anxiety, depression, or cognitive impairment). A help-seeking journey can lead to professional interventions and diagnostic services (formal help), it may result in the use of accessible informal care and self-help, or even in doing nothing at all, as evidenced by low actual help-seeking (Rickwood, 2022). In the case of involuntary treatment or commitment, services can also be provided without a request for help from the individual. However, whenever possible, the use of such direction should be limited, for example by supporting interventions for emerging mental health conditions and providing sufficient attention for recovery from longer-term conditions to prevent acute crises (Patel et al., 2023).

Professional services regularly entail a waiting time since the demand can surpass service capacity. Recent data on waiting times is fragmented, but over 60% of countries considered waiting times to be an issue in mental health services in 2020 (OECD, 2020). For example, Subotic-Kerry et al. (2025) observed that Australian adolescents with anxiety and depression had initiated appointments with more than two treatment providers on average (mostly referred by their GP). The average waiting time was about 100 days but differed between providers, for example 128 days for psychiatrists and 83 days for inpatient units. Less than a third received a waiting time of less than 6 weeks, which is the NHS benchmark. While waiting, mental distress can increase and individuals can experience a lack of support (Subotic-Kerry et al., 2025).

### 2.2 Informal support

In line with the WHO model of optimal care, CoMMA includes informal services as a potential (first) step in help-seeking. This includes **Informal community care**, which refers to services and individuals in the community which are not part of the formal health and welfare system but are more accessible and can play a role in providing support (WHO, 2009). Examples could consist of teachers or online peer support groups (e.g., Morton et al., 2025). Whenever possible, community care should operate in a collaborative care approach where community care providers partner up with mental health specialists, offering supervision, support, and referral pathways (Patel et al., 2023). Informal community care can be offered as a stand-alone service, but can also contribute to supporting individuals on a waiting list or in treatment. Subotic-Kerry et al. (2025) for example indicated that, during their waiting time, adolescents with anxiety and depression were interested in support which was low intensive, non-clinical and communication-based.

**Self-care** has been defined by the WHO (2022; p. 8) as “the ability of individuals, families and communities to promote health, prevent disease, maintain health and cope with illness and disability with or without the support of a health worker”. This includes self-management (e.g., self-administration), self-testing (e.g., self-screening), and self-awareness which includes self-help (WHO, 2022). In the mental health field, research generally focuses on **self-help**, which can be defined as providing yourself with what you need for help, e.g., to solve a problem, end a habit, or learn a skill (Cambridge University Press, n.d.). Self-help can be an effective mental health intervention with several advantages, such as reduced stigma, lower costs, and scalability (Fitzpatrick et al., 2024). It can be performed through bibliotherapy (self-help books; Gualano et al., 2017) but increasingly relies on digital resources (Löchner et al., 2025; McAllister et al., 2020). The first goal of self-help and self-care often consists of finding information, which can occur through various (digital) channels. Although specific digital information sources for health information are not always mentioned, reviews suggest that while older adults rely on traditional health websites, young adults favor social media for health information (Stifjell et al., 2025; Zhao et al., 2022).

Self-help also goes beyond psycho-education and can include self-help tools for behavior change, relaxation and mindfulness, skills acquisition, cognitive restructuring, or mood tracking. However, the digital self-help offer can differ substantially between cultures and languages and evidence-based intervention also require such cultural adaptation. For example, It has been suggested that Western evidence-based psychological interventions can be less valued in faith-based Arab societies, which is evidenced by lower inclusion of evidence-based interventions in Arabic apps compared to Australian apps (Alnaghaimshi et al., 2024). Additionally, while app installations can be high, retention over a longer period of time is very low (illustrated by a 30-day retention of just 3.3% in the study of Baumel et al., 2019). Retention rates and effectiveness of self-help could be improved by offering it in a guided format, where guidance can exist of contacts through e.g., forum, e-mail, phone or perhaps even AI (Gutierrez et al., 2024; Van Daele et al., 2020). However, long-term engagement might not always be needed. Digital self-guided single session interventions (SSI) aim to provide a structured psychological intervention within one interaction (Kaveladze et al., 2025). Although real-world effectiveness needs to be established, evidence-based focused SSI do show promise. Additionally, the content and design of the app (e.g., inclusion of gamification) can promote user engagement and goal achievement (Fitzpatrick et al., 2024). Fitzpatrick et al. (2024) state that the public should also be supported in recognizing effective apps in a domain which is inundated with commercial unhelpful or even harmful approaches. With limited formal regulation from governments, several evaluation initiatives and app repositories have attempted to tackle this challenge, such as the ORCHA (UK) (Hyzy et al., 2024), mHealth Index and Navigation Database (MIND; US; Herpertz et al., 2025), MindApps.dk (Denmark) or Onlinehulp-apps (Belgium) (Van Assche et al., 2024).

In recent years, generative Artificial Intelligence (AI) has also emerged as a new digital resource for help-seeking (Lopez-Lopez et al., 2025). While generative AI has the potential to make health information more accessible, there are substantial concerns about generative AI's ability to engage in human-like interaction and to amplify confirmation bias (Carlbring et al., 2023). Auf et al. (2025) identified three types of AI which can support mental health decision-making: diagnostic and predictive AI, treatment selection AI, and self-help AI. This review observed that AI systems could provide support to actors within (e.g., primary care physician) and outside (e.g., patient, caregiver) formal health, although there was a lack of evidence on implementation and integrative use in care as well as concerns regarding potential biases and engagement.

Informal community care and self-help can lead to accessing formal healthcare services, but this is not necessarily the case. Additionally, the use of these services can be continued while accessing formal healthcare. This corresponds with the Model of Optimal Care where formal mental health services are situated in higher tiers of the pyramid, which are only provided to a smaller part of the population, and self-care should be complementary process (WHO, 2009). The following elements of the CoMMA model draw on the services of trained mental health professionals. While digital mental health can seem like a completely different and distinct field from traditional mental health services, many if not all of acquired professional psychological and social skills apply to both technology-enhanced and face-to-face care. For example, communication should be empathetic and tailored to the target population - taking into account factors such as age, health literacy, and digital literacy - while also upholding high standards of confidentiality, ethics, and privacy. Health literacy refers to literacy and numeracy skills that enable individuals to obtain, understand and appraise health information and use this information to maintain or improve their health (Nutbeam and Lloyd, 2021). Digital literacy “involves the confident and critical use of a full range of digital technologies for information, communication and basic problem-solving in all aspects of life” (UNESCO Institute for Statistics, n.d.). Digital (health) literacy of professionals is also key and often remains a challenge, as digital mental health is often still largely absent in the curricula (Smith et al., 2023). The field would benefit from incorporating digital skills as core competencies in professional training. Additionally, including digital navigators in collaborative care teams can also substantially facilitate implementation.

### 2.3 Diagnostic process

A mental health concern can lead to the initiation of a diagnostic process. Diagnostic assessments for mental health problems and disorders can consist of interviews, screening instruments and questionnaires, observation, studying patient files, and potentially supplementary research (e.g., using tasks or physical examinations). Diagnostic assessments should lead to better insights into the functioning of an individual and may lead to a formal diagnosis according to common diagnostic classification systems such as the International Classification of Diseases (ICD) and Diagnostic and Statistical Manual (DSM). However, such formal diagnostic classification systems have been criticized for not being able to account for heterogeneity within categories, dynamic changes within individuals, and pre-clinical stages of a condition that would benefit from an intervention (Patel et al., 2023).

A diagnostic assessment can take place entirely in a face-to-face format, can be performed completely online, or can be conducted in a blended format where certain activities or tests are performed online or digitally. A face-to-face format generally consists of one or more visits to a professional to complete interviews, diagnostics instruments, or tests. Conclusions can in this case be hampered by a potentially one-time measurement in a fairly artificial setting. A fully online format can accommodate problems in mobility or access, but can also suffer from other limitations. Online symptom checkers have for example shown low diagnostic accuracy and suboptimal triage accuracy (Riboli-Sasco et al., 2023). However, studies have supported the reliability of remote assessment, for example for assessment of executive functions and learning skills in children (Ahmed et al., 2022; Lampis et al., 2024). However, these authors suggest exercising caution when assessing certain types of information, such as reading comprehension. Overview articles have identified a need for more evidence supporting online assessment and attention should be paid to ethical issues, methodological issues (e.g. reliability and validity of AI assessments), technical issues, and the development of training and practice guidelines (Carvalho and Castro, 2025; Geraldo et al., 2024; Salimuddin et al., 2025). Multi-modal approaches including practitioner observations alongside digital assessments can accommodate for these limitations and provide more fine-grained insights.

New technologies have led to advancements in the assessment field. Growing use of smartphones and smartwatches has led to the emergence of contextualized assessment, precision mental health, and use of digital phenotyping. In such approaches, genotypic, clinical, and lifestyle data can be collected in real life through sensors using methodologies such as Ecological Momentary Assessment (EMA), and AI algorithms can use such data to aid in the prediction of mental health complaints and matching of intervention offers (e.g., Lipschitz et al., 2026; Patel et al., 2025). As mentioned above, AI can also contribute to triage.

### 2.4 Assessment of knowledge, skills and attitudes

Before recommending a specific service (following informal care, diagnostic assessment or immediately after an initial request for help) briefly assessing an individual's knowledge, skills and attitudes relating to mental health could be relevant. This information can help to personalize services, which can in turn increase the chances of successful outcomes. Firstly, individuals will likely have some existing **knowledge** regarding (digital) mental health, based on for example educational initiatives, previous experience, or help-seeking experiences. However, knowledge that an individual has gained prior to reaching formal healthcare services (through social media for example), may not always be correct (Karasavva et al., 2025). Therefore, as a professional, it is relevant to assess and promote health literacy and falsify incorrect information.

Secondly, certain **skills** can be required to make use of a proposed treatment, such as health literacy or digital literacy. The knowledge and skills individuals have can also translate into **beliefs and attitudes** that will influence the willingness to use technology in the context of health and well-being. Information on the knowledge, attitudes, and skills, can for example be assessed by questionnaires based on the Technology Acceptance Model (TAM; Tetik et al., 2024) or the Unified Theory of Acceptance and Use of Technology (UTAUT, UTAUT2; Venkatesh et al., 2003, Venkatesh et al., 2012) or other frameworks. However, the relevance of certain determinants can vary depending on context and not all predictors of technology use have received strong empirical support (Blut et al., 2022). Information on knowledge, skills, and attitudes can be used to identify factors which deter from using technology-enhanced services. These factors can subsequently inform intervention selection and implementation, for example for determining a suitable service (in line with matched care) or for addressing existing challenges by providing tutorials or skills training.

Taken together, in addition to diagnostic outcomes (including problem complexity and severity to support intensity-based allocation of services), identifying a suitable mental health service can benefit from (1) reviewing knowledge, skills and attitudes in users, (2) increasing (or correcting) knowledge and skills when needed by providing information about conditions and interventions, (3) assessing user preferences to tailor professional services, and (4) considering equity and inclusion in the approach by tailoring communication and/or providing support when needed. Determining the most suitable service is a joint responsibility for patient and professional, where a patient is empowered as an active partner in intervention selection. GPs or other primary health professionals can be gatekeepers and personal guides to navigate the healthcare system and find a suitable service. However, self-referral has also proved to be an effective (and at times even more effective) route to care and AI-enabled self-referral tools (e.g., Limbic Access) can also support intervention matching, especially for minority groups where this technology can help to reduce stigma and raise awareness of treatment needs (Habicht et al., 2024). Different referral pathways appear to serve different patient populations and have complementary roles in a healthcare system (Bjarke et al., 2025).

### 2.5 Evidence-based mental health service

An evidence-based mental health service can subsequently be offered in a matched care or stepped care (with stratified elements) approach. An individual could be matched to an offer based on available information from an individual's request for help, potential diagnostic assessment outcomes, knowledge, skills, and attitudes. Evidence-based medicine has been defined as “the conscientious, explicit, and judicious use of current best evidence in making decisions about the care of individual patients” (Sackett et al., 1996, pp. 71–72).The use of evidence-based practice and evidence-informed decision-making has been endorsed by e.g., the American Psychological Association (APA Presidential Task Force on Evidence-Based Practice, 2006) and WHO (World health organization, 2021). It must be noted however, that the priorities of research and practice do not always align. Digital programs and technologies such as, for example, extended reality have been subject to a large number of systematic review studies while online consultations have received less research interest (De Witte et al., 2021). On the contrary, online consultations seem to have been implemented in practice to a much greater extent than these other technologies (Van Daele et al., 2025). The mental health system and context of implementation will also limit the available intervention options, for example in stepped care approaches, digital clinics, etc.

A selected mental health service can consist of **face-to-face** therapeutic or counseling sessions in a residential or ambulatory setting. Specialist (short-stay) inpatient facilities are suitable for acute and severe mental health conditions but should be complemented by ambulatory services or community care to provide further care and support (Patel et al., 2023). In line with digital clinics, a treatment plan can also be provided entirely **online** (e.g., online interventions, online consultations or online cognitive behavior therapy (CBT) programs; Andersson and Carlbring (2022) and Mathiasen et al. (2018)). Online services can accommodate for patient preferences, need for anonymous help or practical barriers such as physical distance or mobility problems. Research has suggested that online consultations can be a viable alternative to face-to-face consultations and an effective mental health service, although more controlled studies are warranted (Batastini et al., 2021; Greenwood et al., 2022). Meta-analyses have shown that (therapist-guided) Internet-delivered CBT was equally effective as face-to-face CBT and that it showed long-term efficacy in terms of remission, reliable improvement, and treatment response for many disorders including anxiety, depression, OCD, and PTSD (Andersson et al., 2017; Hedman-Lagerlöf et al., 2023; Zainal et al., 2024). Apart from individual formats, there is also evidence supporting online group therapy (Laurito et al., 2024). Internet-delivered treatments have been proposed to reduce waiting times, increase patient recovery, and potentially also reduce costs given they can offer similar treatment effectiveness (Catarino et al., 2023).

The mental health service can also consist of **blended** or hybrid counseling or therapy, in which face-to-face contact is combined with digital interventions (Lundin et al., 2024). Diel et al. (2025) have shown that in an inpatient context, digital interventions were mostly implemented in the form of aftercare to maintain treatment effects or improve symptoms, although there was an increase in digital inpatient interventions in recent years. Both blended and aftercare implementations have shown positive outcomes in terms of symptom improvement but adherence could be a challenge. While blended therapy is less established than traditional services and has a less extensive research tradition, findings do suggest that individuals hold fairly positive views toward this type of treatment (De Witte et al., 2025). Matching care and tailoring approaches is, however, relevant. A recent systematic review and meta-analysis of digital mental health interventions for university students with mental health difficulties, found that, for anxiety, fully automated interventions may be more effective than guided interventions to reduce symptom severity, while a blended model was more effective for students affected by depression (Madrid-Cagigal et al., 2025). Internet-based and blended interventions have also been suggested to be cost-effective, although more research is needed (Mitchell et al., 2021). Finally, when the request for help does not align with the available interventions offered, individuals should be **referred** to other available services (preferably in close geographic vicinity of the patient). In case of a diagnosed mental illness, pharmaceutical treatment should be considered to complement the psychotherapeutic treatment. Psychosocial interventions have been proven to be effective in combination with pharmacotherapy for disorders such as depression, schizophrenia, and substance use disorder (Cuijpers et al., 2023; McPheeters et al., 2023; Salahuddin et al., 2024).

### 2.6 Technology selection and intervention delivery

When a service includes online or digital components, professionals should select a suitable **technology** as well as the appropriate hardware and software. Several factors can influence modality selection such as availability of technology or the internet, digital literacy skills, pricing (e.g., free applications vs. using an expensive VR headset). AI recommender systems could again play a role in a selection process but the process of modality selection and implementation can also be facilitated by a digital navigator. When the preparatory steps have been completed, the **services can be delivered**. The professional can work together with the patient to address their needs using evidence-based techniques and interventions.

### 2.7 Follow-up

Both during and after service delivery, professionals should provide follow-up and monitor experiences and symptom change. Collectively determining goals and discussing progress facilitates patient empowerment and allows to change the treatment plan when needed. Given that an individual patient data meta-analysis of 29 ICBT trials found deterioration in 5.8% of patients receiving Internet-based Cognitive Behavioral Therapy and 17.4% of those in control conditions, continuous symptom monitoring is essential to detect and potentially reverse a negative treatment trend (Rozental et al., 2017). Follow-up can be facilitated by for example Patient-Reported Outcome Measures (PROMS). PROMS aim to measure outcomes, e.g., symptom change, well-being, goal attainment, from the client perspective (Benson, 2023). To assess change, multiple measurement points are needed per client and a link to the patient's electronic health records is advised. PROMS can be generic or specific to a certain diagnosis or treatment. While PROMS usually consist of extended questionnaires assessing long-term changes, other (digital) monitoring approaches can contribute to short-term and continuous assessment of evolutions and transferal of skills to daily life, for example EMA. Additionally, Patient-Reported Experience Measures (PREMS) can provide insight into client experiences with the mental health service. PREMS are usually conducted anonymously (Benson, 2023). Monitoring of access to and effectiveness of a mental healthcare system should also occur on a systems level to increase accountability (Patel et al., 2023). When healthcare systems collect data from practice to help understand prior experiences and analyze them with appropriate retrospective study designs, such learning healthcare systems have the potential to support better decision making and continuous healthcare improvement (Shiner and Watts, 2021).

After intervention completion, professionals should assess the need for further mental health services. This can lead to the identification of additional mental health concerns followed by new activities (back to the top of the CoMMA model). Alternatively, the services can be concluded and individuals return to their daily routines. It can be relevant to facilitate access to additional resources to prevent relapse and/or promote resilience and a healthy lifestyle (e.g., booster sessions or self-care mental health applications).

## 3 Discussion and conclusion

During the past decades healthcare delivery has tried to adapt to challenges relating to demands, resources, and digital transformation of care. While some healthcare providers and systems have been able to develop into more inclusive and accessible models of healthcare delivery, many others struggle to incorporate innovations and to find their way in the new, community-based or digital landscape (WHO, 2025). The CoMMA model aims to provide a structured yet flexible approach for the provision of evidence-based mental health services, with special attention to the increasing digital transformation of the field. The purpose of the model is to provide guidance to healthcare systems, professionals and trainees in looking at help-seeking processes and shaping the provision of evidence-based psychological services. It hereby aims to complement ongoing societal, regulatory, and economic changes in the healthcare field by providing a conceptual and substantive narrative.

CoMMA is a conceptual and exemplary model, not a compulsory one. While patient journeys can follow the iterative process in the diagram, the model is not meant to be a rigid linear progression that should be adhered to at all costs. In clinical practice, it can be useful to circle back to previous steps, to skip steps or to offer several services in parallel. For instance, if a particular intervention (such as an online service) does not deliver the expected benefits, it may be necessary to move back from the follow-up stage to the diagnostic phase or revisit the assessment of knowledge, skills, and attitudes. It is also important to acknowledge that individuals are embedded in a context and that provided face-to-face or digital interventions mustn't always be oriented toward the individual. Care pathways can include family-focused interventions and mental healthcare can strongly benefit from interventions targeting schools, neighborhoods or communities. Such interventions can also include digital components, for instance a school-based prevention program using a serious game (De Jaegere et al., 2024) or digital social platforms to enlarge the social circle older adults to combat loneliness (Yu et al., 2023).

The CoMMA model adopts central principles from integrated and collaborative care. Therefore, model implementation will require collaboration within primary care but also beyond care organizations and systems. In line with part 1.2. on allocation of healthcare services, referrals could occur in different ways and include self-referral and AI tools. The CoMMA model deliberately does not focus on specific technologies, of which both form and function can quickly change, as evidenced by the way conversational artificial intelligence is currently shaking up the sector (e.g., Bucher et al., 2025). However, technological solutions implemented within this model need to adhere to general regulatory requirements (e.g., from US Food and Drug Administration, EU Medical Device regulation, EU AI act) and important standards concerning privacy, equity, reliability, and interoperability. Care organizations and (digital interventions) can be distinct, unconnected systems, which prevents easy intervention switching and causes administrative burden. Interoperability is an important cornerstone for smooth transitions between CoMMA model elements. Further studies are needed to discuss and propose a technical operationalization of the model.

The current model is a theoretical exercise showing how mental health services can be organized based on current scientific frameworks, policy perspectives, and clinical practice. As such, it is at the moment not actively being used to shape, run or assess services. Successful implementation of a technology-enhanced collaborative care model hinges not only on program elements, but requires a systems approach including attention to digital health regulation, the payment model in mental healthcare, implementation processes and recipient buy-in (Bond et al., 2023; Connolly et al., 2021). Therefore, transferring the CoMMA model to practice requires knowledge of the implementation context, stakeholder involvement and coproduction of concrete services and referral pathways. Improving and diversifying mental health access and interventions (through the adoption of a contextualized CoMMA approach) could have benefits on an individual (e.g., patient mental health), societal (e.g., pressure on the healthcare system), and economic (e.g., technology provider market) level. However, depending on the context, implementation might need to be accompanied by substantial healthcare reforms. Some good practice examples of reimbursement programs already exist (e.g., Schmidt et al., 2024). Identifying the expected costs and benefits and relying on implementation frameworks will be important steps in contextualizing and adopting the CoMMA model.

Existing services could use CoMMA as starting point to optimize processes, implement tools and shape mental healthcare delivery. CoMMA also aims to inspire policy makers to break through siloed funding and services and rethink mental health access pathways. Researchers and implementation scientists could develop future iterations of the model.

## Declaration of competing interest

The authors have no conflicts of interest to declare.
